# Stimuli‐Responsive Particle‐Based Amphiphiles as Active Colloids Prepared by Anisotropic Click Chemistry†

**DOI:** 10.1002/anie.202001423

**Published:** 2020-03-25

**Authors:** Cornelia Lanz, Moritz Schlötter, Nele Klinkenberg, Patricia Besirske, Sebastian Polarz

**Affiliations:** ^1^ Department of Chemistry University of Konstanz Universitätsstrasse 10 78457 Konstanz Germany; ^2^ Institute of Inorganic Chemistry Leibniz-University Hannover Callinstrasse 9 30167 Hannover Germany

**Keywords:** active colloids, core–shell structures, Janus nanoparticles, magnetite nanoparticles, smart materials

## Abstract

Amphiphiles alter the energy of surfaces, but the extent of this feature is typically constant. Smart systems with amphiphilicity as a function of an external, physical trigger are desirable. As a trigger, the exposure to a magnetic field, in particular, is attractive because it is not shielded in water. Amphiphiles like surfactants are well known, but the magnetic response of molecules is typically weak. Vice‐versa, magnetic particles with strong response to magnetic triggers are fully established in nanoscience, but they are not amphiphilic. In this work colloids with Janus architecture and ultra‐small dimensions (25 nm) have been prepared by spatial control over the thiol‐yne click modification of organosilica‐magnetite core–shell nanoparticles. The amphiphilic properties of these anisotropically modified particles are proven. Finally, a pronounced and reversible change in interfacial stabilization results from the application of a weak (<1 T) magnetic field.

## Graphical abstract

**Particle‐based amphiphiles**: Ultra‐small Janus particles (JPs) have been prepared using magnetite/organosilica particles in a click reaction with spatial control. The JPs show a pronounced and reversible magnetic response when applying a weak magnetic field to a dispersion which results in a difference in the interfacial energy.

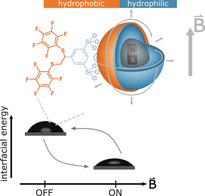

The prototypes for amphiphilic compounds originate from molecular chemistry, like surfactants or block‐copolymers. Unique properties and important applications result from the combination of reluctant parts such as hydrophobic and hydrophilic in one molecular entity. The latter is responsible for interfacial activity within amphiphiles and the capability for complex self‐organization.^1^ The degree of interfacial activity is constant for the vast majority of molecular systems, because it is difficult to alter the molecular configuration to such an extent, amphiphilic properties may become stimuli‐responsive. However, amphiphiles that can be turned on and off by demand would be highly interesting.

The higher sensitivity of nanoparticles towards external triggers is only one argument, why particle‐based amphiphiles have raised interest.^2^ While only few publications can be found for a literature survey on particle‐based amphiphiles, an impressive amount of publications exist for particles with so‐called Janus design.^3^ The classic examples for a Janus particle (JP) involve colloidal beads with the two hemispheres differing in chemical composition.^4^ Therefore, the successful preparation of a JP requires some sort of symmetry break. One can roughly differentiate between two preparation approaches. The JP can be assembled from two individual and chemically different species, which are joint together. This approach is also described as compartmentalization.^5^ The alternative strategy is a partial (!) modification of the surface of the original particle. This can be achieved by restricting the accessibility of one hemisphere by immobilization of the particle(s) on a surface or by imbedding them in a matrix like a polymer or wax.^6^ Clearly, these methods work better the larger and less mobile the particles are. This is one of the reasons, why JPs smaller than 50 nm in diameter are hard to find in literature.^7^ However, thinking of stimuli‐responsive, particle‐based amphiphiles, minimization of the size/mass of the JPs is desirable. A smaller size and mass would increase the sensitivity towards the external trigger and thus, lead to more versatile active colloids than currently known.^8^ As amphiphiles operate in water, it would also be an advantage, if the system could react to a magnetic field, because it is not shielded in an aqueous electrolyte.^9^ The latter arguments define the target system for the current work: A nano‐sized (<50 nm) JP‐based amphiphile, which reacts on an externally applied magnetic field. The blueprint of the target JPs is shown in Scheme 1, and an overview about the particles presented in the current study is given in Figure S1 in the Supporting Information.

**Scheme 1:**
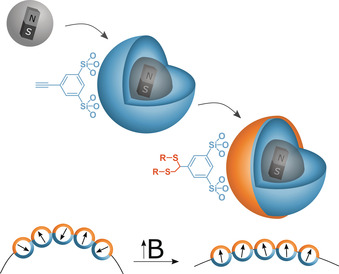
Stimuli‐responsive particle‐based amphiphiles. Silica encapsulated magnetite nanoparticles are covered with a shell of alkyne‐modified organosilica. The anisotropic modification of the alkyne groups by click chemistry leads to JPs. Amphiphilicity originates from suitable selection of R (=hydrophobic group). The application of a magnetic field is expected to orient the magnetic dipoles of the superparamagnetic cores, which induces an additional force. This could have an influence on the way, the particles stabilize the interface.

Two preliminary achievements are important to mention. Others have published impressive work on the encapsulation of single‐domain magnetite nanoparticles in silica.^10^ The advantage of a silica shell is, it is chemically robust, the particles become dispersible in polar solvents, and, last but not least, many routes are known for the modification of silica surfaces.^11^ Our group could recently establish an organosilica system, which is suitable by modification using the so‐called click chemistry.11a, 12 The advantages of click chemistry have been discussed in several review articles and could not be better represented than by the Nobel Prize in 2001.^13^

Monodomain magnetite (Fe_3_O_4_) nanoparticles (NPs) with a diameter of 9.8 nm (polydispersity index (PDI)=6 %) were prepared according to a method published by Sturm et al. in 2017.^14^ The unambiguous characterization is summarized in Supporting Information Figure S2. Next, we adapted the method published by Ding et al. for encapsulating the Fe_3_O_4_‐NPs with a shell of silica.11c The resulting particles are shown in Supporting Information Figure S3. The thickness of the SiO_2_ shell can be minimized by controlling the amount of tetraethyl orthosilicate (TEOS) added to the synthesis mixture. The smallest value of the SiO_2_ layer is only 1 nm.

The alkyne‐containing shell was prepared by a sol‐gel process with a novel, phenylacetylene‐bridged silsesquioxane precursor (**4**) shown in Scheme 2 (see also experimental section in the Supporting Information). We start from the phenylbromide‐bridged sol‐gel precursor (**1**), which can be transformed to multiple other groups exploiting the full potential of aromatic substitution chemistry.11a, 11b, 12a, 12b, 15 Here, the bromide is substituted by a trimethylsilyl‐protected acetylene (**2**) catalyzed by copper(I) iodide/ 1,1′‐bis(diphenylphosphino)ferrocenedichloropalladium(II).^16^ The trimethylsilyl group is removed from (**3**) by treatment with AgNO_3_ leading to the new sol‐gel precursor (**4**). (**4**) was characterized by ^1^H‐, ^13^C‐ and ^29^Si‐NMR spectroscopy (Supporting Information Figure S4). The NMR spectra prove the purity of the compound, in particular the presence of only one ^29^Si‐signal at −62.93 ppm. The signals at 3.08 ppm/84.51 ppm and 77.36 ppm (^1^H, ^13^C) show the presence of the acetylene function. The successful synthesis is also demonstrated by electrospray ionization mass spectrometry (ESI‐MS) (Supporting Information Figure S4). Hydrolysis and condensation lead to the phenylacetylene containing organosilica material (AlkySil). The chemical nature of the material was characterized by ^13^C‐ and ^29^Si‐NMR, ATR‐IR, FT Raman spectroscopy and thermogravimetric analysis (TGA) (see Supporting Information Figure S5).

**Scheme 2:**
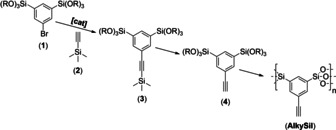
Reaction sequence leading to organosilica materials modified by a bridging phenylacetylene group. R=iso‐Pr.

The final, core–shell–shell particles (Figure 1 a) were prepared as described in detail in the experimental section in the Supporting Information. The size and morphology of the synthesized Fe_3_O_4_/SiO_2_/AlkySil particles were characterized by TEM (see Figure 1 b). The particle diameter increased from 20.8 nm (Fe_3_O_4_/SiO_2_ particles, PDI=8 %) to 22.9 nm (PDI=5 %). The successful formation of the AlkySil shell is confirmed by the different imaging contrast seen in TEM. In TEM the particles seem to be agglomerated. However, this can be excluded by analytical ultracentrifugation (AUC) measurements. The resulting particle size distribution curve (*d*_h,max_=26.1 nm) is shown in Figure 1 c, and is consistent with the TEM data when compared to isolated particles. Complete characterization of the particles is summarized in Supporting Information Figure S6.

**Figure 1:**
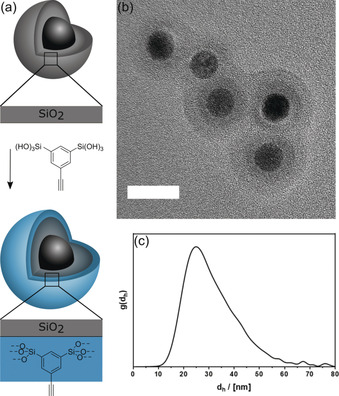
a) Synthesis and architecture of Fe_3_O_4_/SiO_2_/AlkySil core–shell–shell particles. b) TEM image; scalebar=20 nm. c) Particle‐size distribution function derived from AUC measurement with *ρ*(Fe_3_O_4_/SiO_2_/AlkySil)=2.5 g cm^−3^.

The alkyne‐functionalized core–shell–shell particles can be further modified via two different types of click reactions, the Huisgen cycloaddition and the thiol‐yne click reaction.^17^ For proof of concept, both types of reactions have been applied. The modification of the particles with the dye Cumarin‐343 was achieved by the Huisgen cycloaddition (Supporting Information Figure S1,7). The advantage of using a dye is, the success of the surface modification can be shown very easily by optical methods. Furthermore, NPs modified by fluorescence tags are promising for investigating their behavior as active colloids by particle tracking microscopy.

The photochemical thiol‐yne variant is demonstrated using mercaptoundecanoic acid (Figure 2). As it is not possible by electron microscopy to visualize the presence of the organic constituents directly, Zn^2+^ ions were coordinated to the carboxylic acid groups to increase the imaging contrast. A bright field STEM image is shown in Figure 2 b. Compared to the Fe_3_O_4_/SiO_2_/AlkySil particles (Figure 1 b), a thin, dark rim at the exterior surface can be clearly identified. An energy‐dispersive X‐ray spectroscopy (EDX) linescan (see Figure 2 c) confirms that this dark rim consists of Zn. Additional data is shown in Supporting Information Figure S8. The reference experiment, the treatment of Fe_3_O_4_/SiO_2_/AlkySil particles with Zn^2+^ (Supporting Information Figure S9), proves that the coordination of the metal at the surfaces is due to the successful click reaction with mercaptoundecanoic acid.

**Figure 2:**
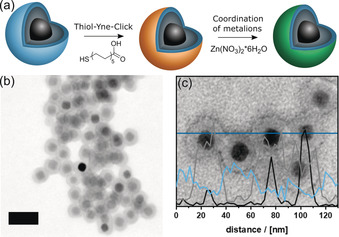
a) Synthesis of Fe_3_O_4_/SiO_2_/AlkySil/Zn particles. b) Bright field STEM image of Fe_3_O_4_/SiO_2_/AlkySil/Zn particles; scalebar 50 nm. c) EDX linescan of Fe_3_O_4_/SiO_2_/AlkySil/Zn particles with Fe linescan (black), Si linescan (grey) and Zn linescan (blue), dark blue: position of the linescan.

The second advantage using the photochemical variant of the thiol‐yne reaction is that particles deposited as a monolayer on a surface, which is immersed in a solution containing mercaptoundecanoic acid, can easily be radiated only from one side (Figure 3 a). A representative SEM image of such a monolayer is shown in Supporting Information Figure S10. The additional ZnO‐layer (50 nm thickness) on the silicon wafer facilitates the detachment of the particles after click‐modification. The outcome of the described experiment was analyzed and the results are shown in Figure 3 b,c. It can be seen that the dark rim indicating the presence of Zn is not isotropic in space anymore. Only a segment of 1/4‐1/3 has been modified now with mercaptoundecanoic acid, obviously. The partial modification can also be confirmed by EDX‐measurements (Supporting Information Figure S11). As expected, the content of Zn^2+^ is lower for the anisotropic particles (Figure 3) compared to the isotropic particles (Figure 2).

**Figure 3:**
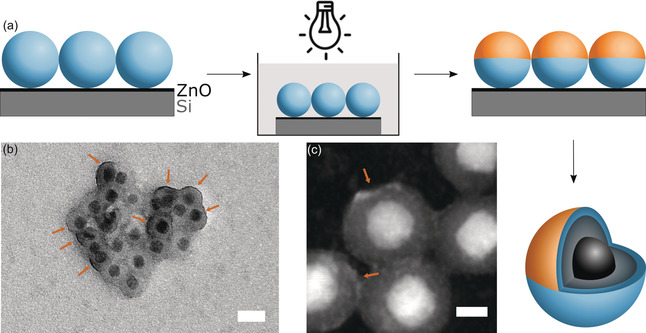
a) Synthesis of Janus particles: Monolayer assembly of Fe_3_O_4_/SiO_2_/AlkySil particles; irradiation and photochemical thiol‐yne reaction; removal of particles from surface. b) TEM image of Janus Fe_3_O_4_/SiO_2_/AlkySil/a‐Zn particles; scalebar 25 nm. c) Dark‐field STEM image; scalebar 10 nm, arrows indicating the anisotropic presence of Zn.

After successfully proving the preparation of ultra‐small Janus nanoparticle, we can (anisotropically) modify the particles with alternative clickable compounds like pentafluorothiophenol to change the amphiphilicity of the JPs by clicking the hydrophobic molecule on one hemisphere whereas the other hemisphere is still unmodified (Figure 4 a).

**Figure 4:**
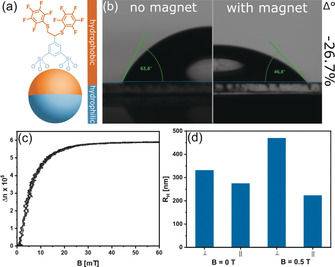
Contact angle determination using the pentafluorothiphenol‐modified JPs (a) for sessile drop method of a JP dispersion drop on a glass substrate (b). Left image shows the drop in absence of a magnetic field compared to a magnet (<1 T) placed beneath the substrate (right). CME measurement (c) and DLS results (d) of the JPs at *B*=0 and 0.5 T perpendicular and parallel to the magnetic field.

The existence of the pentafluorothiophenol entity can be proven by IR and EDX spectroscopy (Supporting Information Figure S12). The corresponding JPs were prepared using the methodology described above. For demonstration of the amphiphilic properties of the JPs, the pendant drop method was used and the liquid‐vapor surface tension (*γ*_lv_=20.4 mN m^−1^) was calculated (see Supporting Information Figure S13). However, more interesting is, if and how the amphiphilicity changes in the presence of a magnetic field. Experiments were performed using the sessile drop method on a glass substrate (Figure 4 b, control experiments in Figure S14). The particles are used in a way very similar to a surfactant. The particles are dispersed in the aqueous phase and are, thus, expected to occupy the liquid‐air interface (Scheme 1). A magnet (<1 T) is placed beneath the substrate and the difference in the contact angle *Θ* is recorded. Surface tensions solid–gas (constant), liquid–gas and solid–liquid enter *Θ*. Thus, the spreading of the drop is indicative for a change in the interfacial energy. As nothing has changed, except for exposure to the magnet, this change in surface energy has to originate in a difference in amphiphilicity of the JPs (Scheme 1). The phenomenon can be described as follows: The magnetite cores of the JPs are superparamagnetic. Thus, in absence of an external magnetic field, the moments are randomly distributed. In the presence of a magnetic field, the domains become oriented resulting in a force on the particles (see also Supporting Information Figure S7). Because one interpretation of the surface tension is the force per distance acting at the interface, the additional, magnetic influence can eventually change *γ*.

One also has to consider, there are gradients in the magnetic field applied in the experiment described in Figure 4 b. Because particles migrate in such a gradient (Supporting Information Figure S7), it cannot be excluded that there are also local changes of the composition of the interface. Migration does not occur in a homogeneous magnetic field. Instead, rotation happens. The reaction to a homogeneous magnetic field was probed by determination of the Cotton Mouton effect (CME), which is the emergence of birefringence caused by a magnetic field. The CME curve (Figure 4 c) show a rapid and strong answer of the dispersion of the JPs exposed to a weak field (0.05 mT). In addition, dynamic light scattering (DLS) indicates the presence of aggregates of the JPs (Figure 4 d). The diffusion coefficient and the resulting hydrodynamic radius R_H_ were determined parallel and perpendicular to the field. For *B*=0 T both values are almost equal, which speaks for spherical aggregates like vesicular structures. A distinct anisotropy is found for *B*=0.5 T, with the extension of the aggregates almost doubled in direction of the field. This speaks for a deformation and/or alignment of the aggregates.

Compared to the literature, for example, normal magnetite/silica core/shell structures, our system shows further advantages such as an easy way to the anisotropic modification via a photochemical click reaction leading to Janus NPs, without the necessity for imbedding of the particles, amphiphilic behavior and a much smaller nanoparticle size, which is advantageous for maximization of response to the external magnetic field and active properties.6c, 11c Starting from core–shell–shell nanoparticles, we showed that the thiol‐yne click reaction can be used for the spatially resolved modification leading to a Janus architecture. The anisotropic modification using hydrophobic moieties leads to particle‐based amphiphilic properties. Addressing the magnetite core by an external field changed the inter‐particle interaction, which had a direct influence on the degree of amphiphilicity.

Up to this point, we could only obtain JP particles, which differ regarding the hydrophobic segments. It seems that the surface coverage of the NPs on the substrate used for the photochemical reaction is a key factor. A perfect monolayer would guarantee a much more homogeneous way of anisotropic modification. The adjustment of the extension of the segments is even more demanding. If one manages to deposit the particles in such a way, they are fully isolated, the modified zone would most likely be enlarged. In essence, the step of depositing the particles on a substrate prior to click modification needs to be optimized in the future. Nevertheless, the system presented herein is not only a versatile platform for new particle‐based amphiphiles, considering click chemistry will allow to modify the surfaces with almost any group and the remaining hemisphere of the JPs (Scheme 1) can still be modified differently.

## Conflict of interest

The authors declare no conflict of interest.

## Supporting information

As a service to our authors and readers, this journal provides supporting information supplied by the authors. Such materials are peer reviewed and may be re‐organized for online delivery, but are not copy‐edited or typeset. Technical support issues arising from supporting information (other than missing files) should be addressed to the authors.

Supplementary
