# Supplementary material for: Stimuli‐Responsive Particle‐Based Amphiphiles as Active Colloids Prepared by Anisotropic Click Chemistry
Source: Angew Chem Int Ed Engl. 2020 Mar 25;59(23):8902–6. doi: 10.1002/anie.202001423 (PMC7318572; doi:10.1002/anie.202001423)
Supplement: Supplementary file 1 — Supplementary [file ANIE-59-8902-s001.pdf]

Angewandte Chemie
Eine Zeitschrift der Gesellschaft Deutscher Chemiker

Supporting Information

Stimuli-Responsive Particle-Based Amphiphiles as Active Colloids Prepared by Anisotropic Click Chemistry**

*Cornelia Lanz, Moritz Schlötter, Nele Klinkenberg, Patricia Besirske, and Sebastian Polarz**

anie_202001423_sm_miscellaneous_information.pdf

Supporting Information

Table of Contents

Experimental 3

Synthesis of 1,3-Bistri(isopropoxysilyl)-benzene-5-acetylene (**4**) 3

Preparation of AlkySil material 3

Preparation of Fe_3O_4/SiO_2 core-shell particles 4

Preparation of Fe_3O_4/SiO_2/AlkySil core-shell-shell particles 4

Preparation of isotropic-clicked Fe_3O_4/SiO_2/AlkySil/Cumarin particles 4

Preparation of isotropic-clicked Fe_3O_4/SiO_2/AlkySil/Zn particles 4

Preparation of anisotropic-clicked Fe_3O_4/SiO_2/AlkySil/a-Zn particles 4

Preparation of anisotropic-clicked Fe_3O_4/SiO_2/AlkySil/a-F-thiol particles 5

Analytical methods 5

Results 7

Experimental

Synthesis of 1,3-Bistri(isopropoxysilyl)-benzene-5-acetylene (**4**)

To a mixture of Pd-DPPF (0.2 mmol, 0.1 eq), DPPF (0.27 mmol, 0.15 eq) and CuI (0.35 mmol, 0.2 eq) in 13.3 mL THF and 6.6 mL NEt_3, 1,3-Bistri(isopropoxysilyl)-5-bromobenzene (**1**) (1.8 mmol, 1 eq) was added. The reaction mixture was degassed for 15 min and trimethylsilylacetylene (28.9 mmol, 16.3 eq) was added before capping the microwave vial. The microwave was heated to 110°C over 20 min, the temperature was held for 45 min before cooling down to 55 °C. The obtained precursor 1,3-Bis(isopropoxysilyl)-benzene-5-trimethylsilylacetylene (**3**) was filtrated and purified via column chromatography. ^{1}H-NMR (400 MHz, $CDCl_3$): δ(ppm) = 7.93 (t, J = 1.2 Hz, 1H, p-arom. CH); 7.80 (d, J = 1.2 Hz, 2H, o-arom. CH); 4.24 (hept, J = 6.1 Hz, 6H, OCH); 1.20 (d, J = 2.1 Hz, 36H, CH_3); 0.26 (s, 9H, Si-$(CH_3)_3$). ^{13}C-NMR (100 MHz, $CDCl_3$): δ(ppm) = 141.2 (p-arom. CH); 139.9 (o-arom. CH); 132.1 (arom. C-Si); 121.9 (<u>C</u>-C≡C); 105.9 (C-<u>C</u>≡C); 93.9 (C-C≡<u>C</u>); 65.5 (OCH); 25.5 (CH_3); 0.1 (Si-$(CH_3)_3$). ^{29}Si-NMR (79 MHz, $CDCl_3$): δ(ppm) = -17.91 (C-Si-$(CH_3)_3$); -62.69 (C-Si-O). HR-ESI-MS: m/z = 605.3043, (simul. 605.3047) [M+Na^+]; deviation: 0.3 ppm.

To deprotect precursor (**3**) a solution of $AgNO_3$ (1.73 mmol, 2 eq) in 20 mL aceton (317.22 eq), 0.9 mL dest. H_2O (60 eq) and 2.09 mL pyridine (30 eq) was produced. 0.5 g precursor (**3**) (0.86 mmol, 1 eq) was mixed with the solution under vigorous stirring and heated to 40 °C for 40 h. For purification AgCl was precipitated by addition of brine and precursor (**4**) was extracted with Et_2O. After removal of the solvent and further purification with column chromatography a colourless oil was obtained. ^{1}H-NMR (400 MHz, $CDCl_3$): δ(ppm) = 7.97 (t, 1.2 Hz, 1H, p-arom. CH); 7.84 (d, 1.2 Hz, 2H, o-arom. CH); 4.25 (hept, 6.1 Hz, 6H, OCH); 3.08 (s, 1H, C-C≡C-H); 1.20 (d, 2.1 Hz, 36H, CH_3). ^{13}C-NMR (100 MHz, $CDCl_3$): δ(ppm) = 141.7 (p-arom. CH); 140.2 (o-arom. CH); 132.5 (arom. C-Si); 121.0 (<u>C</u>-C≡C); 84.5 (C-<u>C</u>≡C); 77.4 (C-C≡<u>C</u>); 65.7 (OCH); 25.6 (CH_3). ^{29}Si-NMR (79 MHz, $CDCl_3$): δ(ppm) = -62.93 (C-Si-O). HR-ESI-MS: m/z = 533.2722 (simul. 533.2725) [M+Na^+]; deviation: 0.5 ppm.

Preparation of AlkySil material

0.3 g precursor (**4**) were dissolved in 1 mL ethanol and with 50 µL 0.1 M HCl prehydrolysed for 2 h. By addition of 50 µL conc. ammonia the condensation started. Afterwards the mixture was transferred into a syringe and closed with parafilm overnight. The yellow material was obtained by drying the gel under standard conditions.

Preparation of Fe_3O_4/SiO_2 core-shell particles

A total of 5.0 g Igepal CO-520 dissolved in 110 mL cyclohexane was sonicated for 15 min before adding 10 mL (3.4 mg/mL in cyclohexane) Fe_3O_4 cores and 0.9 mL conc. ammonia. Under continuous stirring 0.5 mL tetraethylorthosilicat (TEOS) were added with 50 mL/h rate of addition. The reaction was stopped by addition of 30 mL methanol and purification by centrifugation in ethanol. The Fe_3O_4/SiO_2 core-shell particles were redispersed in ethanol.

Preparation of Fe_3O_4/SiO_2/AlkySil core-shell-shell particles

For the prehydrolysation, 40 mg precursor **(4)**, 1.0 mL n-propanol and 50 µL 0.1 M HCl were stirred for 2 h. A total of 20 mg Fe3O4/SiO2 particles dispersed in 140 mL ethanol were stirred and the prehydrolysed precursor **(4)** was added via a syringe pump with 0.1 mL/h rate of addition. The particles were purified by centrifugation in ethanol.

Preparation of isotropic-clicked Fe_3O_4/SiO_2/AlkySil/Cumarin particles

A total of 4.9 mg Tetrakis(acetonitrile)copper(I)hexafluorophosphate and 5.0 mg Cumarin-343-azide were solved in 1.0 mL ethanol and added to 5 mL of 1.0 mg/mL Fe_3O_4/SiO_2/AlkySil particles in ethanol. The reaction mixture was stirred for 24 h and afterwards washed several times by centrifugation in ethanol.

Preparation of isotropic-clicked Fe_3O_4/SiO_2/AlkySil/Zn particles

A total of 125 mg mercaptoundecanoic acid and 1 mg Benzildimethylketal (DMPA) were added to 3 mg Fe_3O_4/SiO_2/AlkySil particles in 3 mL ethanol in a quartz tube. The UV lamp was turned on for 1 min. The obtained clicked particles were washed several times in ethanol. Afterwards Zn ions were coordinated by addition of 30 mg $Zn(NO_3)_2$*$6H_2O$ to the particle dispersion and heated to 70°C for 5 min. The resulting Fe_3O_4/SiO_2/AlkySil/Zn particles were washed by centrifugation in ethanol.

Preparation of anisotropic-clicked Fe_3O_4/SiO_2/AlkySil/a-Zn particles

For the assembly, 120 µL of a 0.8 mg/mL Fe_3O_4/SiO_2/AlkySil particles dispersion in ethanol were dried on a 2x2 cm Si substrate with a 50 nm amorphous ZnO layer. The substrate with the assembled particles was placed in a petri dish containing a solution of 125 mg mercaptoundecanoic acid and 0.5 mg Benzildimethylketal (DMPA). The UV lamp was positioned directly above the petri dish and turned on for 1 min. The obtained particles were removed from the substrate by addition of 150 µL 0.01 M HCl and purified by centrifugation and redispersed in ethanol. Afterwards, Zn ions were coordinated by additon of 15 mg $Zn(NO_3)_2$*$6H_2O$ to the particle dispersion and heated to 70°C for 5 min. The resulting Fe_3O_4/SiO_2/AlkySil/a-Zn particles were washed by centrifugation in ethanol.

Preparation of anisotropic-clicked Fe_3O_4/SiO_2/AlkySil/a-F-thiol particles

For the assembly, 120 µL of a 0.8 mg/mL Fe_3O_4/SiO_2/AlkySil particles dispersion in ethanol were dried on a 2x2 cm Si substrate with a 50 nm amorphous ZnO layer. The substrate with the assembled particles was placed in a petri dish containing a solution of 10 µL pentafluorothiophenol (F-thiol) and 0.5 mg Benzildimethylketal (DMPA). The UV lamp was positioned directly above the petri dish and turned on for 1 min. The obtained particles were removed from the substrate by addition of 150 µL 0.01 M HCl and purified by centrifugation and redispersed in ethanol.

Analytical methods

For the microwave synthesis a microwave from Anton Paar Monowave 300 and 30 mL microwave tubes with cap were used. NMR spectra were measured on a Varian Unity INOVA 400 MHz spectrometer. MAS-NMR spectra were obtained with a Bruker DRX 400 spectrometer with a spinning frequency of 10 kHz. ESI-MS spectra were recorded with Bruker micrOTOF II. TGA measurements were obtained with a Netzsch STA F3 Jupiter. SEM images were acquired with a Zeiss Gemini 500. Raman spectra were acquired using a Raman station 400 from Perkin Elmer. X-ray diffraction was performed with a Bruker AXS D8 advance diffractometer using Cu-K_α radiation. IR spectra were obtained with a Perkin-Elmer Spectrum 100. HR-TEM images, EDX spectra and linescan were acquired with a JEOL, JEM 2200FS at an accelerating voltage of 200 kV. UV/Vis spectra were acquired using an Agilent Cary60. The fluorescence spectra were measured with a PicoQuant FT300. AUC measurements were performed with an Optima SL-I analytical centrifuge (Beckman-Coulter, Palo Alto, CA, USA) with Rayleigh interference in a titania measuring cell (Nanolytics, Potsdam, Deutschland) and centrepiece with 12 mm optical waylength. The field- and temperature-dependant SQUID measurements have been acquired using a Quantum Design MPMS XL 5 magnetometer. The surface tensiometry and contact angle were measured at 20 ± 1 °C using a Krüss drop shape analysis DSA1 apparatus. The instrument calculates the surface tension and contact angle with spatial coordinated of the drop shape and size. Measurements of the particle dispersion in water were taken on the same drop with and without a magnet on a glass substrate. The used NdFeB (cube, 10 mm, magnetic field density of ~ 1 T) was positioned 1 mm from the bottom of the drop. The measurement apparatus is a combination of two classical dynamic light scattering (DLS) systems and a birefringence detection setup. The sample is placed in an electro magnet and illuminated with a laser (HeNe 632nm, 10mW).
One DLS detection optic is aligned at a scattering angle of 9°, with the scattering vector parallel to the magnetic field. The second DLS detection optic is aligned with the same scattering angle but the scattering vector is perpendicular to the magnetic field. The

scattered light is picked up by mono-mode fibers and detected with single photon detectors (ALV SO-SIPD) that are connected to a hardware correlator (Flex02-12D/C from correlator.com). The intensity autocorrelation functions are analyzed separately, by fitting a mono-exponential relaxation. By the use of the Stokes-Einstein relation, the directional diffusion coefficients yield the respective hydrodynamic radius. The birefringence is detected from the transmitted beam. Enhanced detection sensitivity is achieved by the use of a photoelastic modulator (PEM II/FS84 from Hinds Instruments), two Glan-Thompson polarizers (aligned at +-45° with respect to the magnetic field) and a Lock-In amplifier (Stanford Research SR830-DSP). The value of birefringence is measured by compensation of the signal with a pockels cell. For a more detailed description, see e.g. [doi:10.1016/0378-4363(77)90800-2].

Results

Figure S1. Overview of the modification of the Fe_3O_4/SiO_2/AlkySil particles.

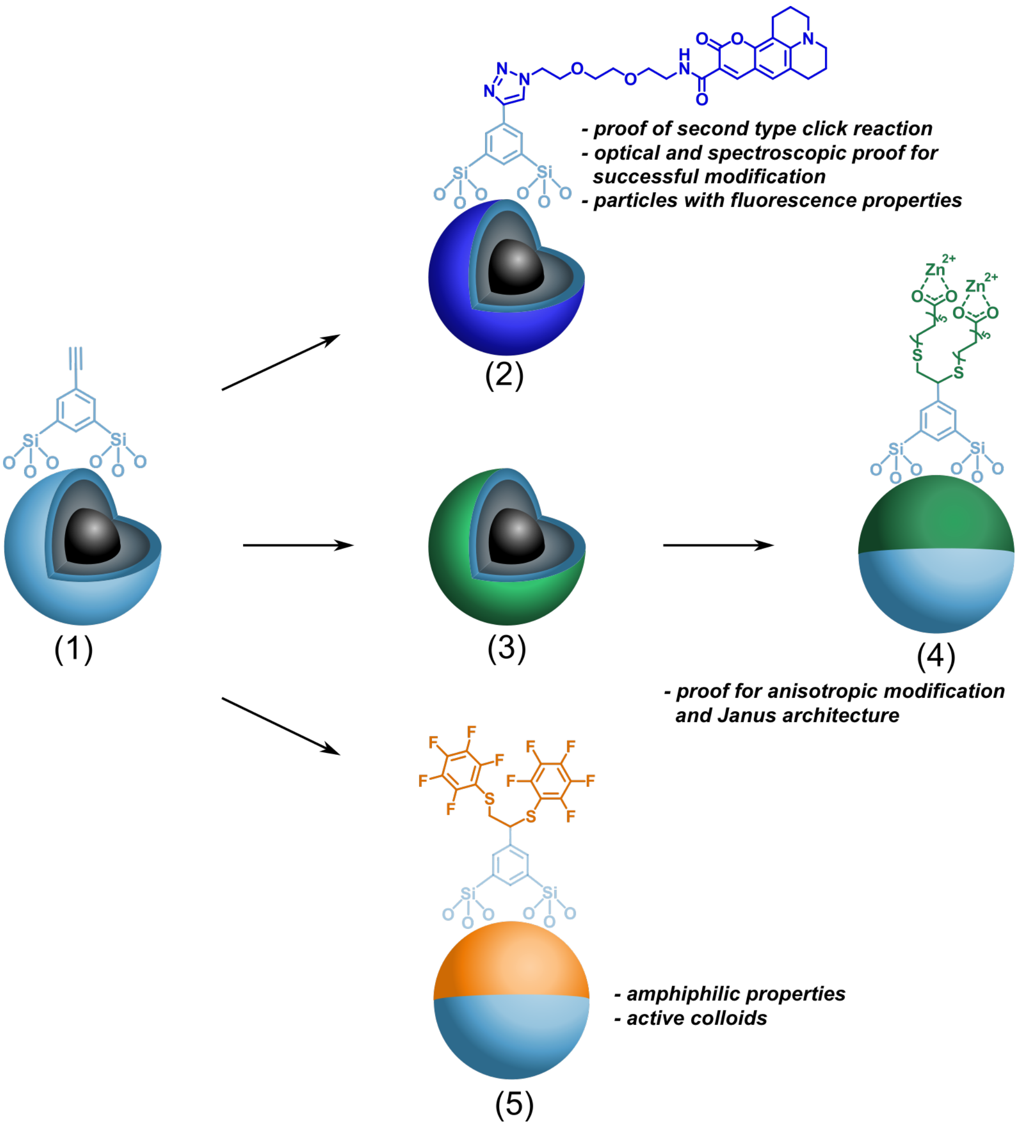

Starting from the Fe_3O_4/SiO_2/AlkySil particles (particle type (1)) two types of click reactions are possible for further modifications. Particle type (2) shows the isotropic Cumarin-343-azide-modified particles via the Cu-catalyzed Huisgen cycloaddition. The other particles are modified via the photochemical Thiol-Yne click reaction. The modification with mercaptoundecanoic acid and the coordination of Zn ions (particle type (3) and (4)) enables the imaging of the modification whereas particle type (5) shows amphiphilic behaviour with the hydrophobic-clicked pentafluorothiophenol.

Figure S2. Analytical data for the magnetite particles.

(a)

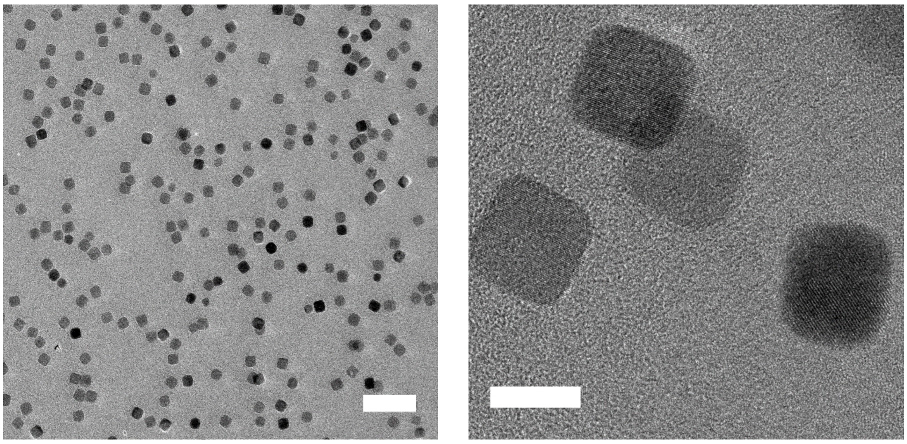

TEM-images (left: scalebar 50 nm, right: scalebar 10 nm).

(b)

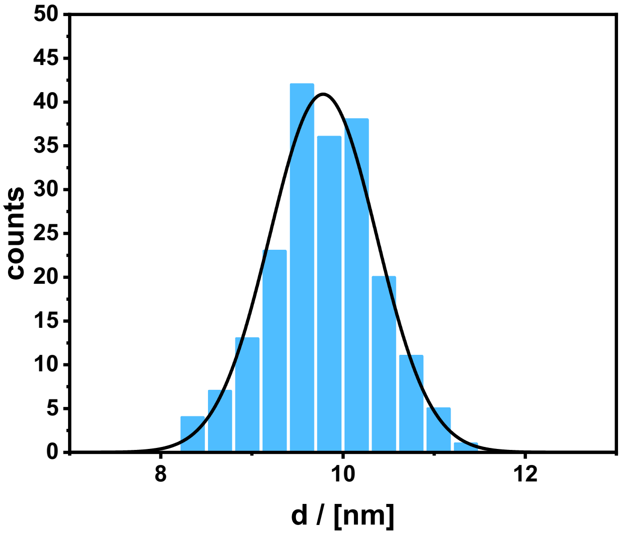

Histogram counted from TEM-images, d = 9.78 nm, PDI = 6.0%.

(c)

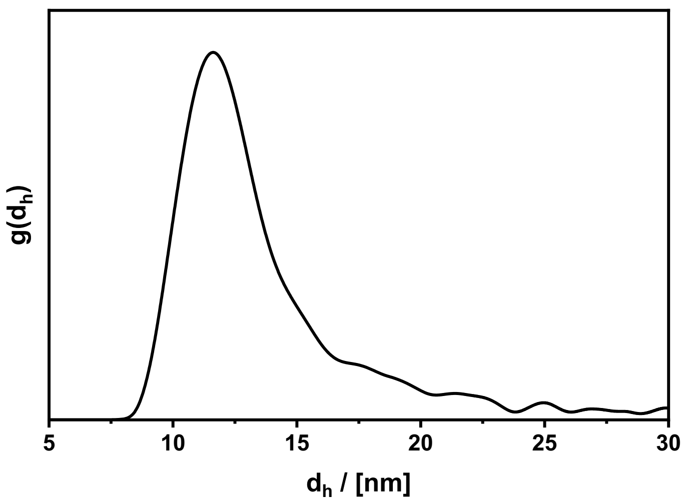

AUC measurement, d_h = 11.6 nm.

(d)

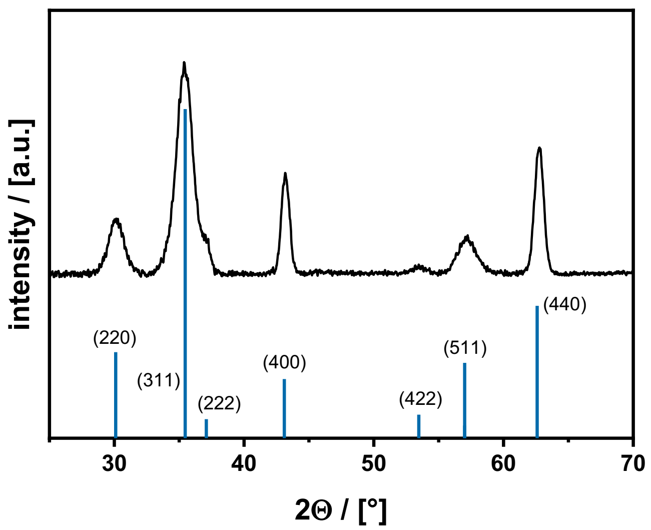

PXRD data (blue: magnetite reference, black: magnetite particles).

(e)

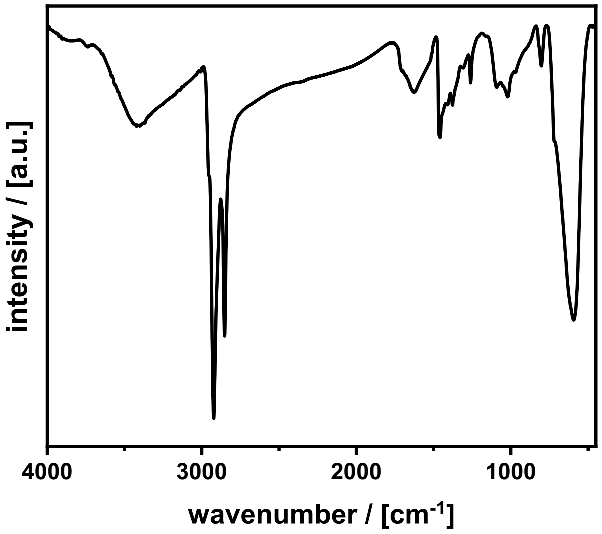

Wavenumber (cm^{-1})	vibration
~ 3400	H_2O (KBr matrix)
2770-2960	CH_3 streching
1630	CO streching
580	FeO streching

IR spectrum.

(f)

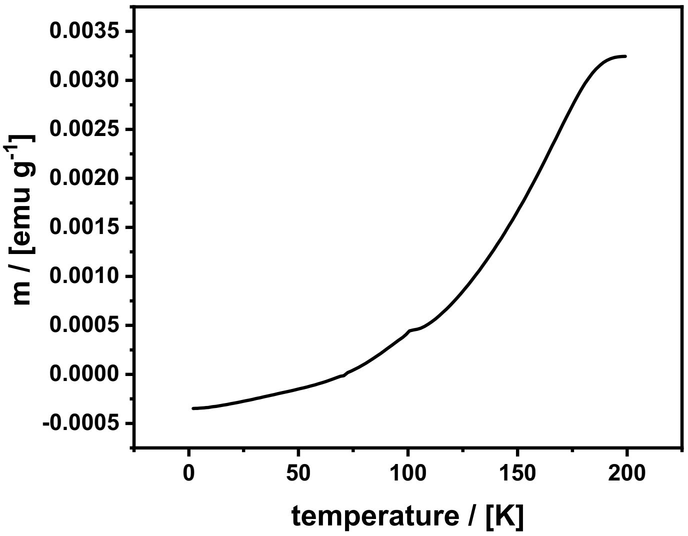

Temperature-dependent SQUID measurement, blocking temperature T_B = 100.7 K.

(g)

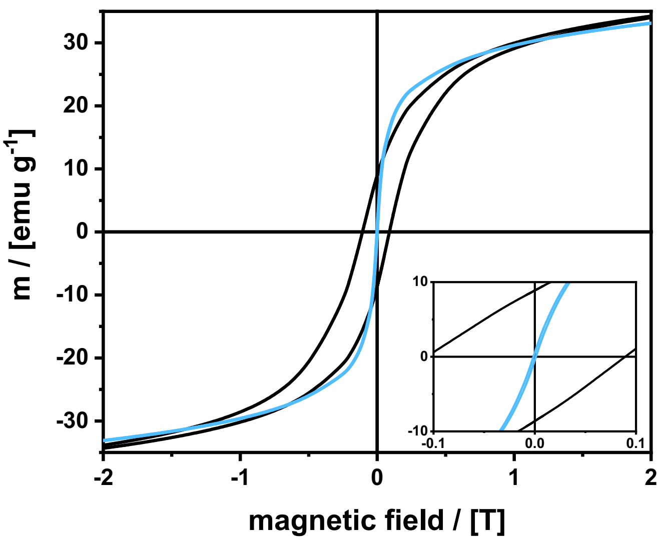

Field-dependent SQUID measurement, blue: measurement at 4 K, m = 39.7 emu/g, ferromagnetic behaviour; black: measurement at 300 K, m = 38.0 emu/g, superparamagnetic behaviour.

Figure S3. Fe_3O_4/SiO_2 core-shell NPs.

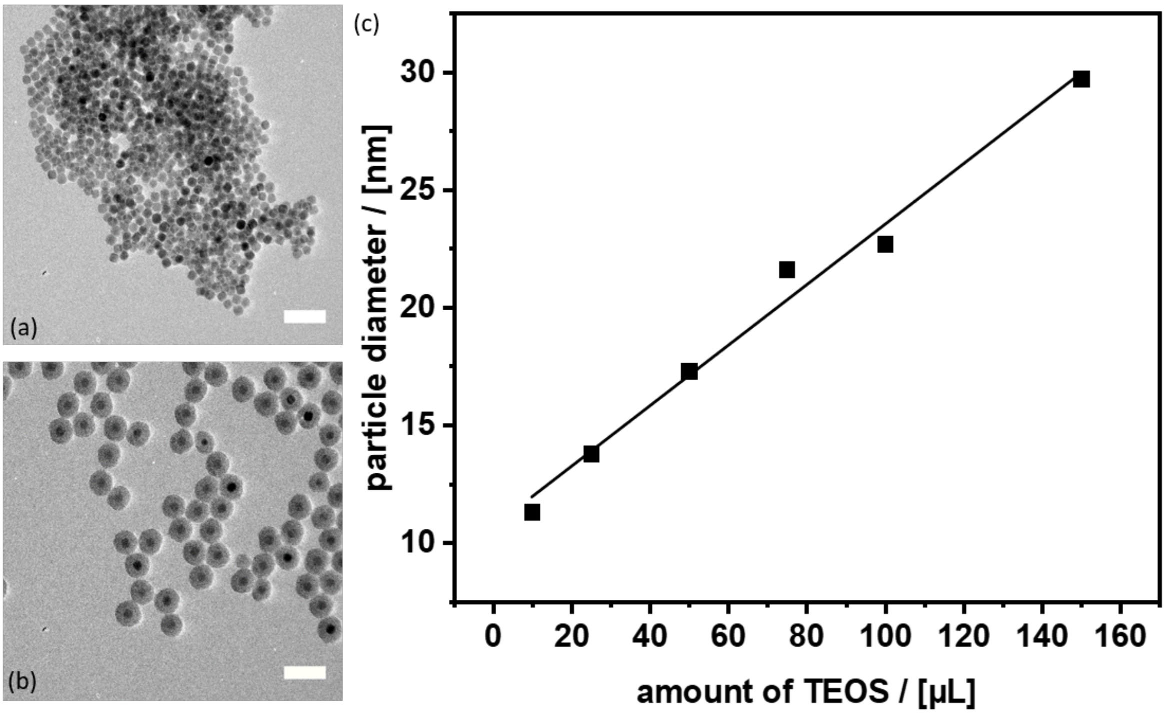

TEM micrographs of Fe_3O_4/SiO_2 core-shell particles with thin (a) and thick shell (b); scalebars = 50nm. Correlation of particle diameter with amount of TEOS used during synthesis (c).

(d)

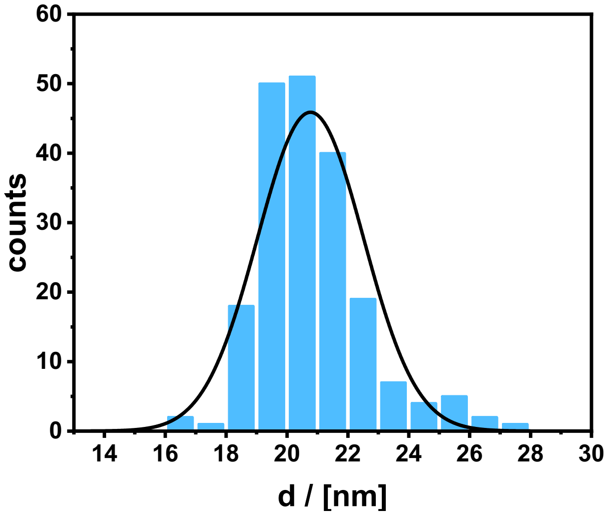

Histogram of the used Fe_3O_4/SiO_2 particles counted from TEM-images, d = 20.8 nm, PDI = 8.0%.

(e)

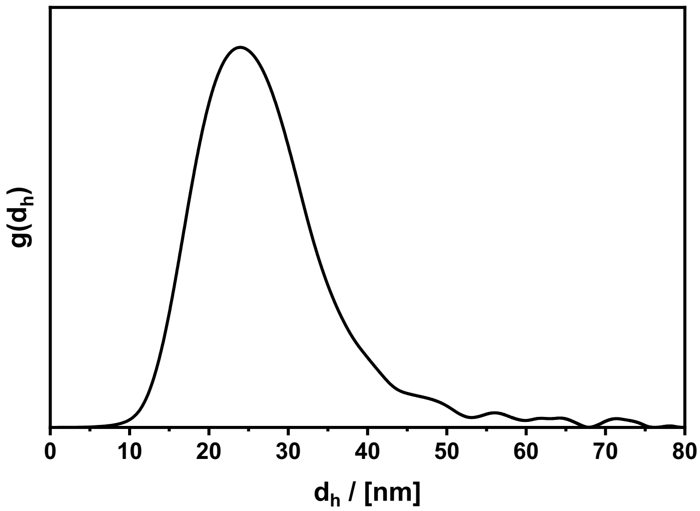

AUC measurement of the used Fe_3O_4/SiO_2 particles, d_h = 23.6 nm with a particle density of ρ = 3.1 g/cm^3.

(f)

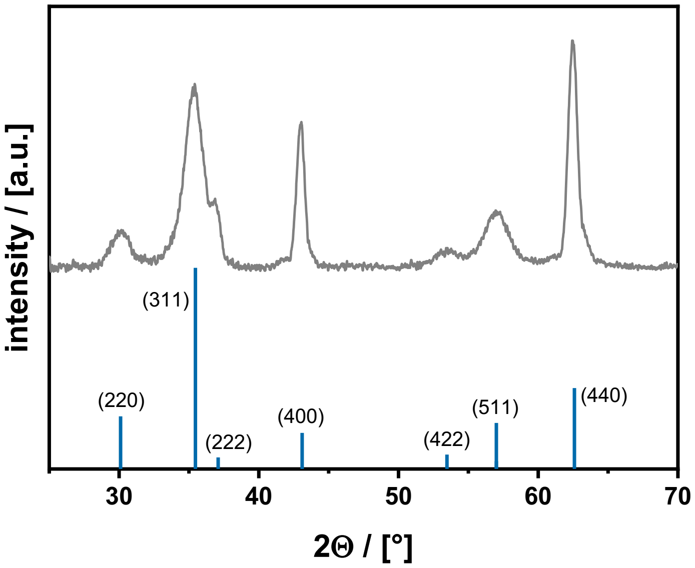

PXRD data (blue: magnetite reference, grey: Fe_3O_4/SiO_2 particles).

(g)

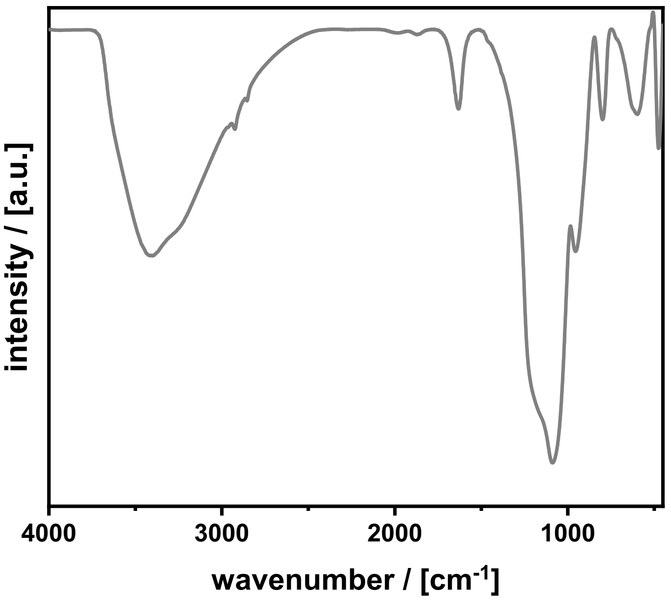

Wavenumber (cm^{-1})	vibration
~ 3400	H_2O (KBr matrix)
2770-2960	CH_3 streching
1630	CO streching
1080	SiO streching
580	FeO streching

IR spectrum of Fe_3O_4/SiO_2 particles.

(h)

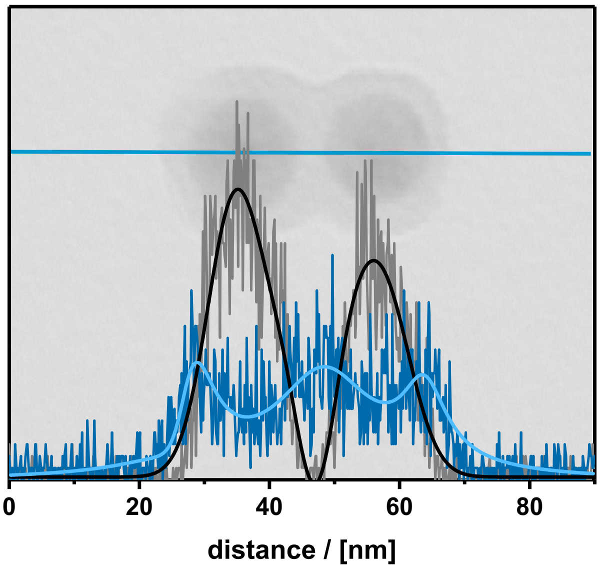

EDX linescan of Fe_3O_4/SiO_2 particles; measured: Fe (grey) with Gauss-fit (black) and Si (blue) with Pearson VII-fit (light blue).

Figure S4. Analytical data for the sol-gel precursor (**4**).

(a)

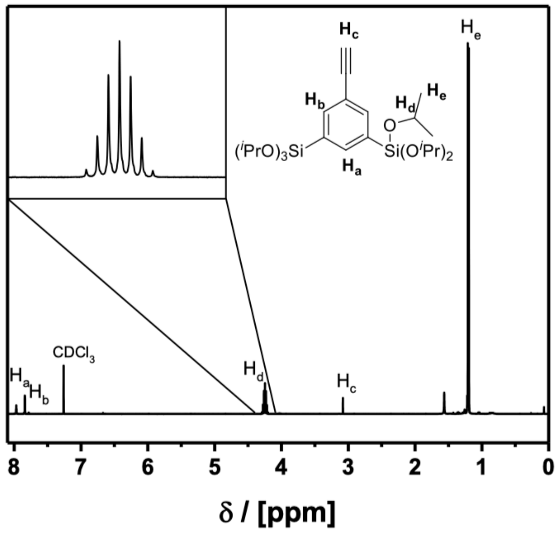

^{1}H-NMR spectrum in $CDCl_3$.

(b)

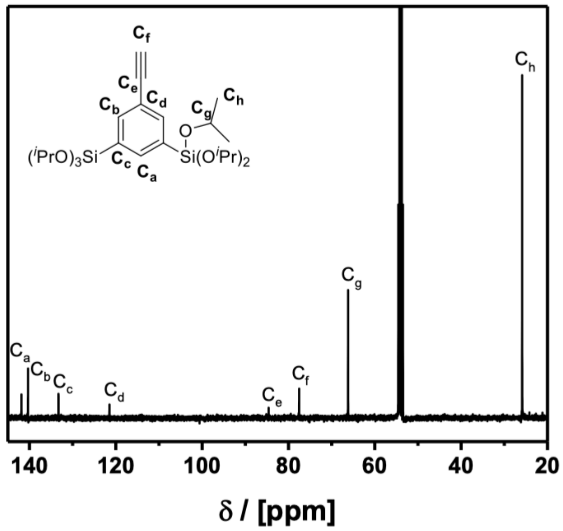

^{13}C-NMR spectrum in $CDCl_3$.

(c)

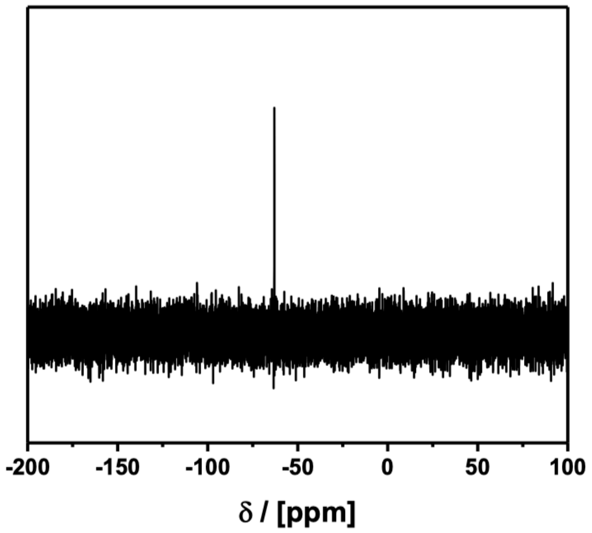

^{29}Si-NMR spectrum in $CDCl_3$.

(d)

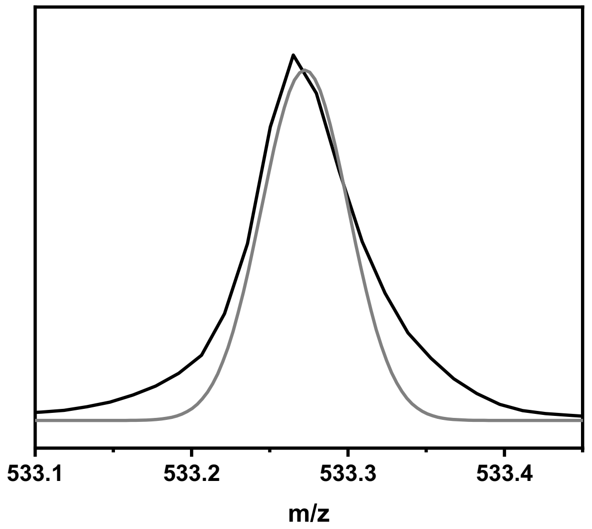

ESI-MS pattern for [M+Na$^+$]; measured (black): m/z = 533.2722 (simul. (grey): 533.2725) [M+Na$^+$]; deviation: 0.5 ppm.

Figure S5. Analytical data for the organosilica material (**AlkySil**).

(a)

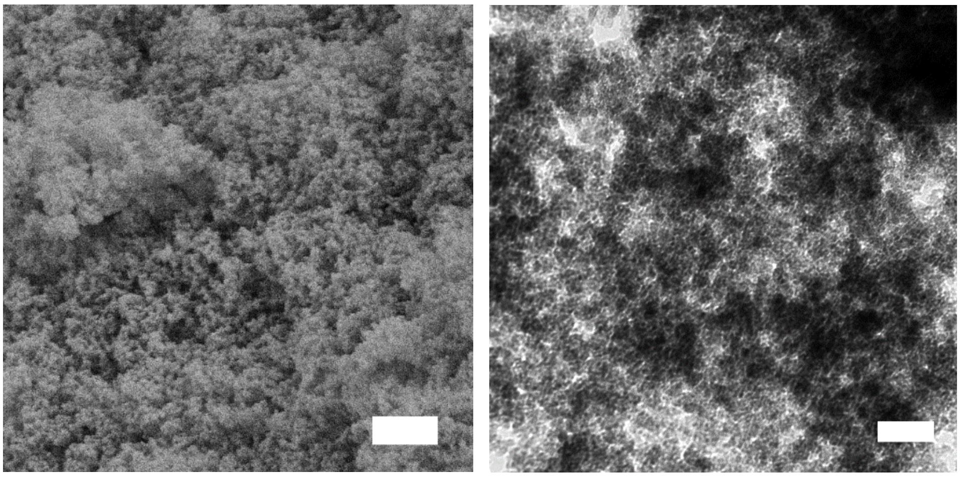

SEM (left, scalebar 500 nm) and TEM (right, scalebar 50 nm) image of AlkySil-material.

(b)

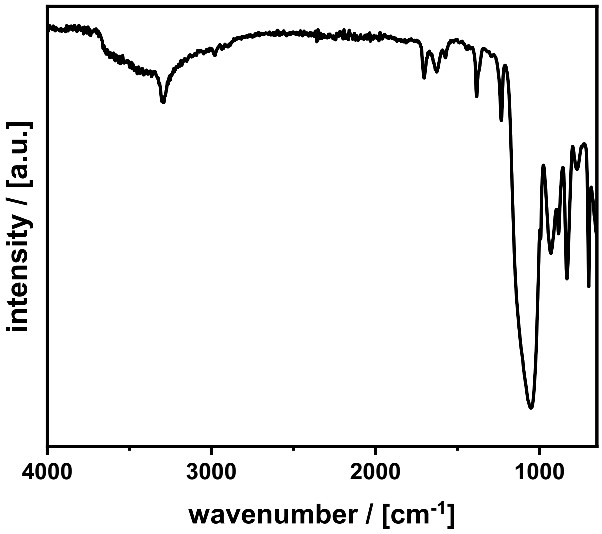

Wavenumber (cm^{-1})	vibration
3295	CH valence
1030	SiO streching
840	Ring deformation

ATR-IR spectrum.

(c)

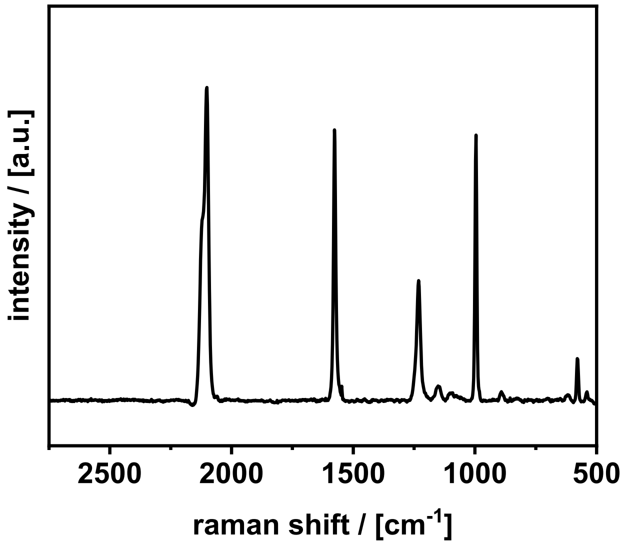

Raman shift (cm^{-1})	vibration
2101	C≡C
1230	
1575	aromatic ring
994	
550	Si-O-Si

Raman spectrum.

(d)

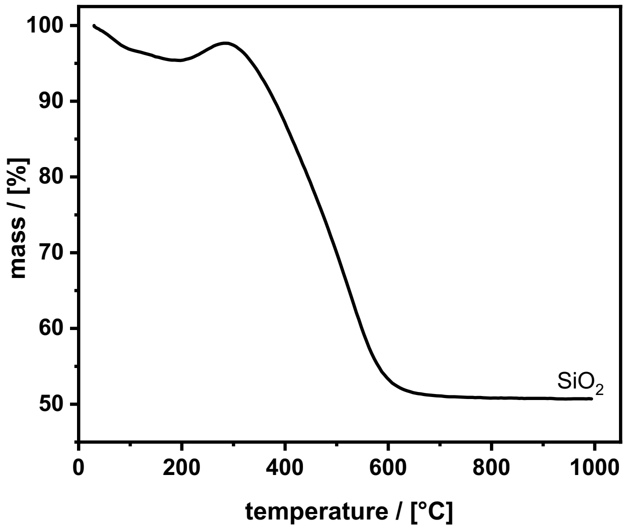

TGA curve, organic amount 49.1%

(e)

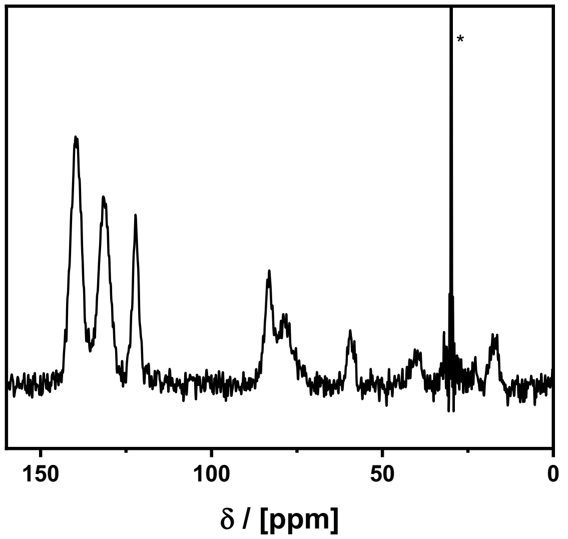

^{13}C-MAS-NMR spectrum.

δ(ppm) = 139.3 (p-arom. CH), 131.4 (o-arom. CH), 122.2 (arom. C-Si), 82.9 (C-C≡C), 79.0 (C-C≡C), 59.4 (C-C≡C), 40.4 (OCH), 16.9 (CH3).

(f)

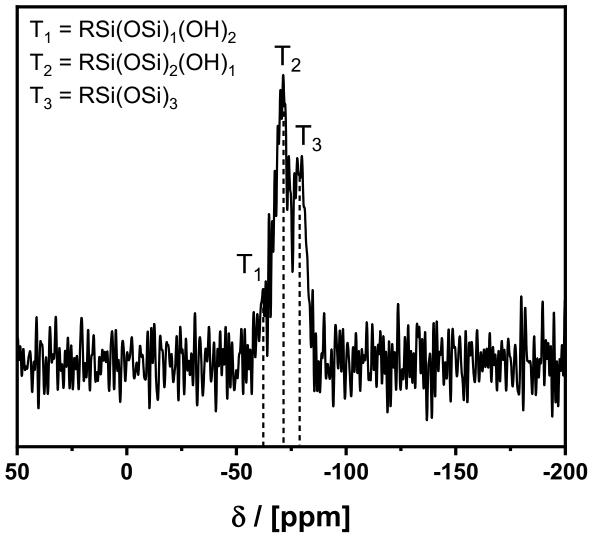

^{29}Si-MAS-NMR spectrum.

Figure S6. Analytical data for the Fe_3O_4/SiO_2/AlkySil core-shell-shell particles.

(a)

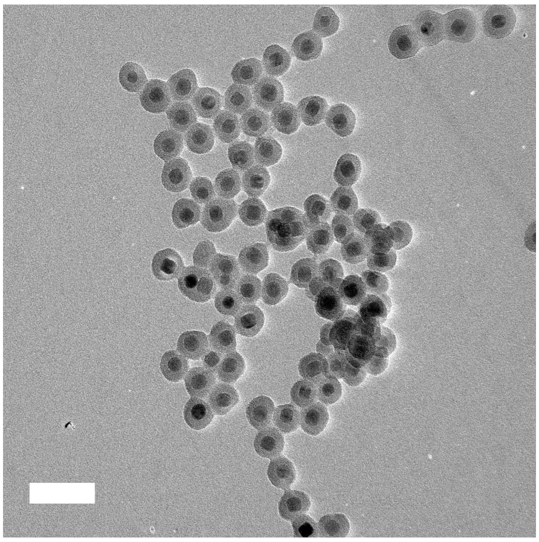

TEM image overview of Fe_3O_4/SiO_2/AlkySil particles, scalebar 50 nm.

(b)

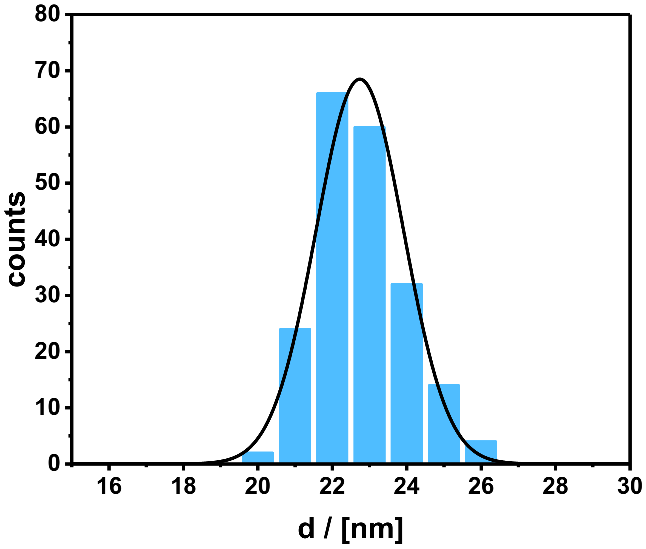

Histogram of the Fe_3O_4/SiO_2/AlkySil particles counted from TEM-images, d = 22.9 nm, PDI = 5.0%.

(c)

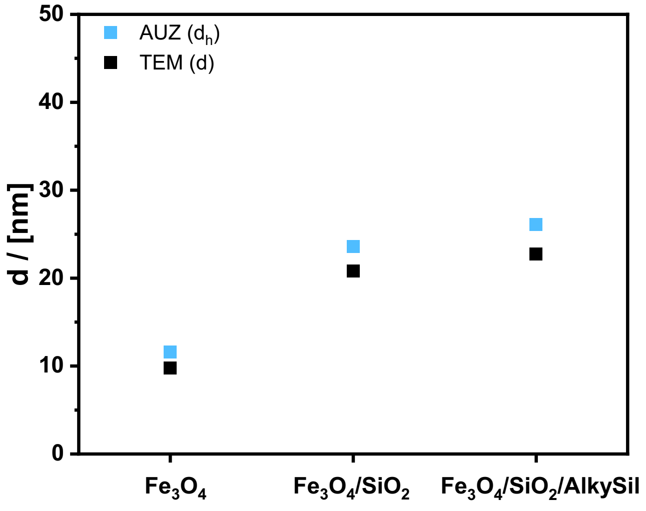

Comparison of the diameter of the different particle types from TEM images (black) and AUC measurements (blue).

(d)

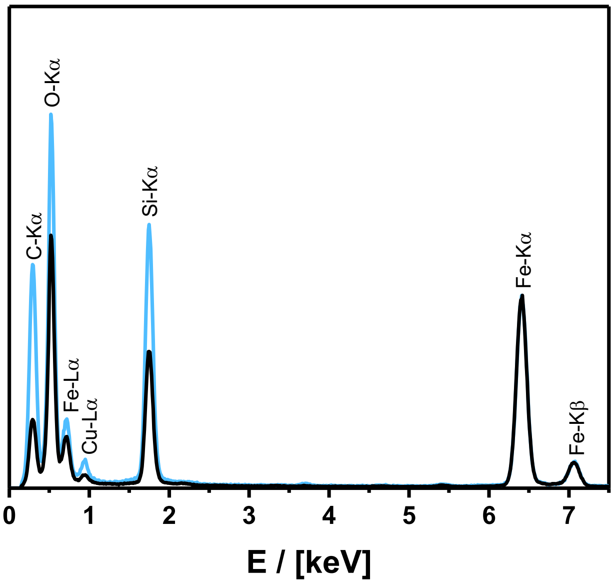

EDX spectrum of Fe_3O_4/SiO_2 (black) and Fe_3O_4/SiO_2/AlkySil particles (blue). Referenced to Fe-signal; Fe_3O_4/SiO_2 particles less Si than Fe; Fe_3O_4/SiO_2/AlkySil particles more Si than Fe.

(e)

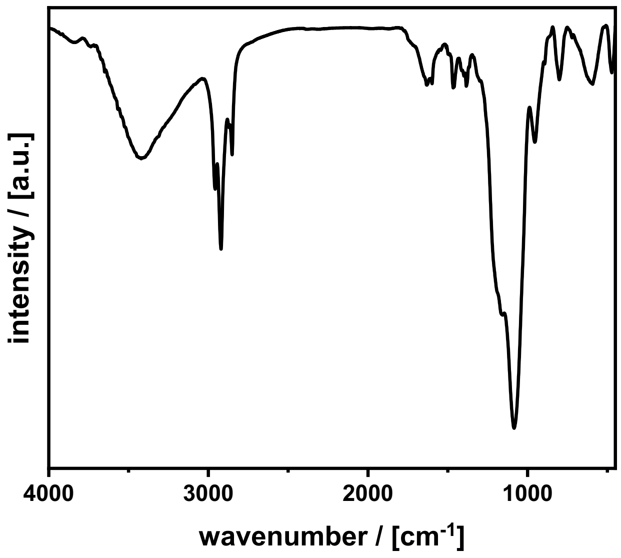

Wavenumber (cm^{-1})	vibration
~ 3400	H_2O (KBr matrix)
2770-2960	CH_3 streching
1630	CO streching
1080	SiO streching
580	FeO streching

IR spectrum of Fe_3O_4/SiO_2/AlkySil particles.

(f)

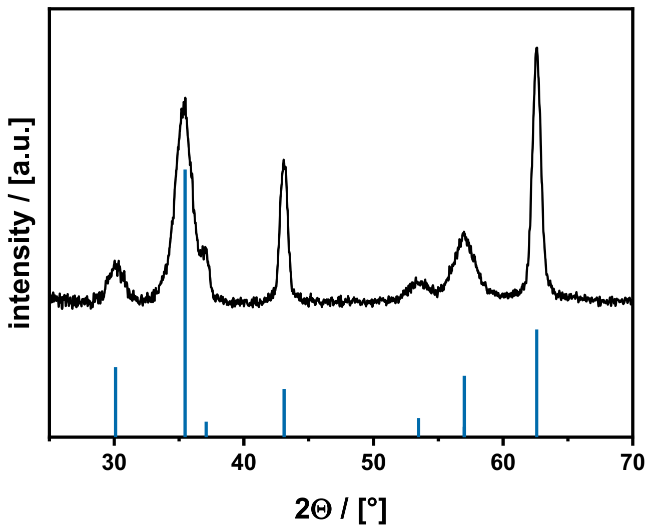

PXRD data (blue: magnetite reference, grey: Fe_3O_4/SiO_2/AlkySil particles).

(g)

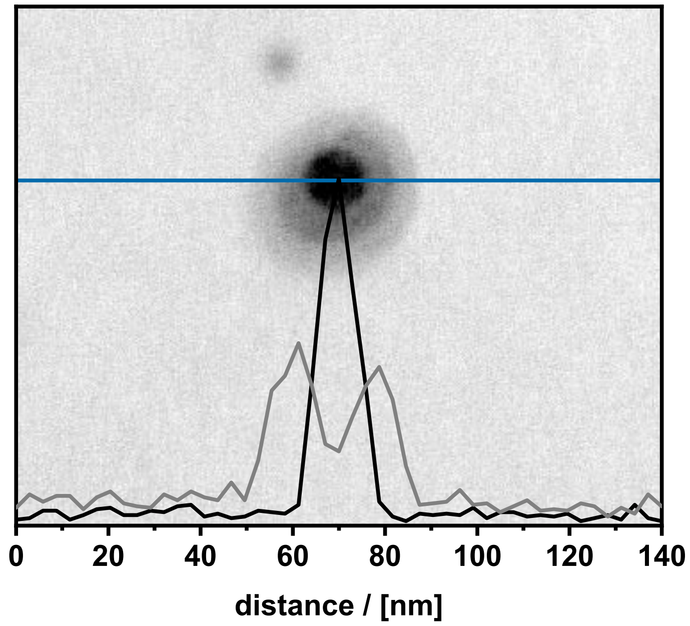

EDX-Linescan of the Fe_3O_4/SiO_2/AlkySil particles; Fe (black) and Si (grey).

(h)

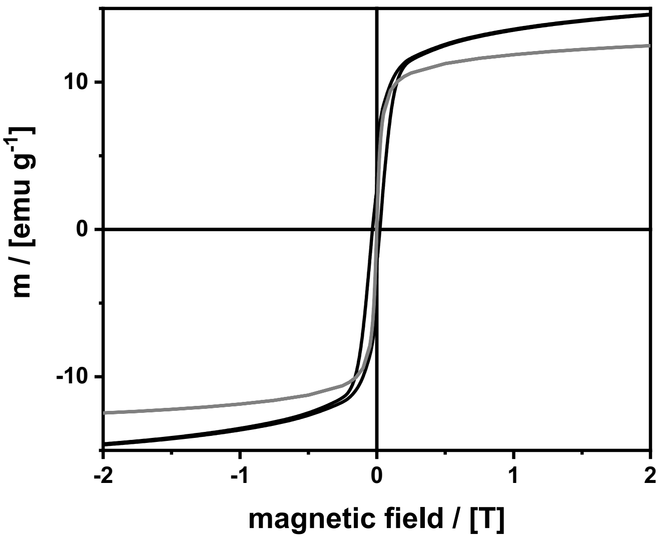

Field-dependant SQUID measurement Fe_3O_4/SiO_2/AlkySil particles at 1.8 K (black) and 300 K (grey). Fe_3O_4/SiO_2/AlkySil particles show same behaviour as the magnetite particles (see S1).

(i)

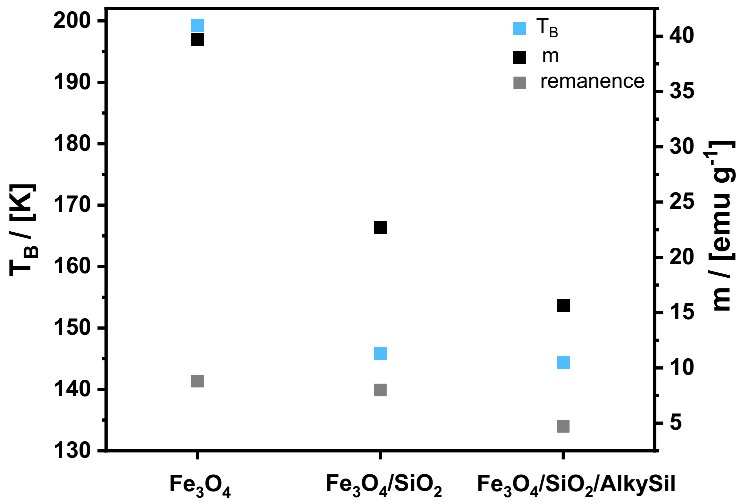

	T_B / [K]	m / [emu g^{-1}]	R / [emu g^{-1}]
Fe_3O_4	199.2	39.7	8.8
Fe_3O_4/SiO_2	145.9	22.7	8.0
Fe_3O_4/SiO_2/AlkySil	144.28	15.6	4.7

Comparison of the Blocking temperature (T_B, blue), magnetic moment (m, black) and remanence (grey) of Fe_3O_4, Fe_3O_4/SiO_2 and Fe_3O_4/SiO_2/AlkySil particles. With introducing non-magnetic silica as first shell the blocking temperature, magnetic moment and remanence decreases and further decreases with the second shell consisting of AlkySil.

Figure S7. Analytical data for the isotropic click modification of the Fe_3O_4/SiO_2/AlkySil particles by the copper-catalyzed 1,3-dipolar Huisgen cycloaddition.

(a)

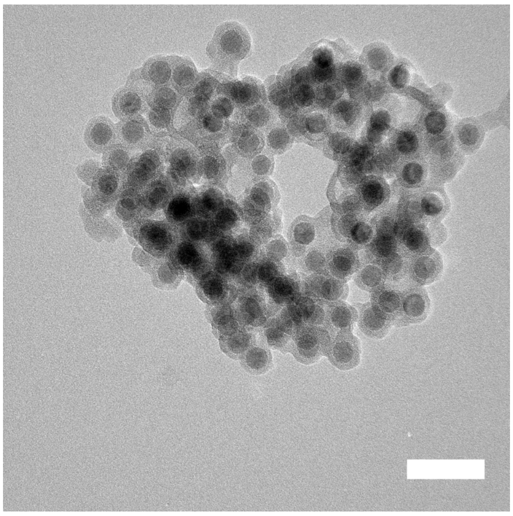

TEM image of Cumarin-343-clicked Fe_3O_4/SiO_2/AlkySil particles, scalebar 50 nm. Morphology is unchanged after click reaction.

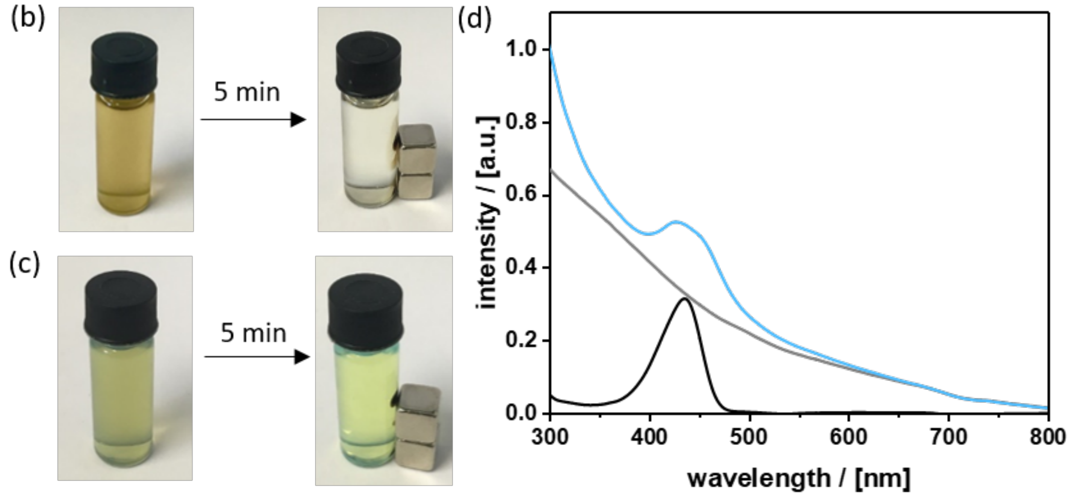

(b) Magnetic separation of Fe_3O_4/SiO_2/AlkySil/Cumarin particles. (c) Magnetic separation of negative control with dissolved dye. Due to chemically-bound dye the solution of the dispersion is colourless (b) whereas the negative control (c) is yellow coloured after magnetic separation of the particles. (d) UV/Vis spectra of Cumarin-343-azide (black), Fe_3O_4/SiO_2/AlkySil/Cumarin particles (blue) and negative control (grey). The particles show high scattering at low wavelengths because of the magnetite cores, as well as an absorption band measured at 437 nm (blue), which conforms to the absorption band of the dye Cumarin-343-azide (black).

The negative control experiment (click reaction with Fe_3O_4/SiO_2 particles) shows that the dye is covalently-bound and not just adsorbed on the surface of the particles.

(e)

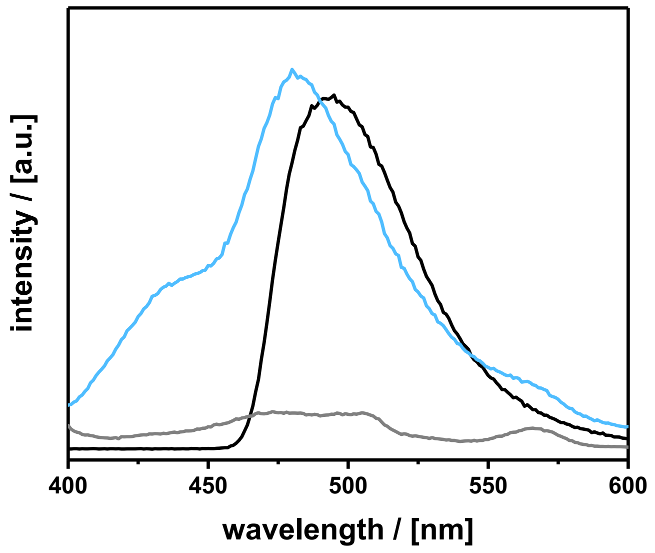

Fluorescence spectra of Fe_3O_4/SiO_2/AlkySil/Cumarin particles (blue), Cumarin-343-azide (black) and negative control (grey).

Figure S8. Analytical data for the isotropic click modification of the Fe_3O_4/SiO_2/AlkySil particles by the photochemical Thiol-Yne reaction with mercaptoundecanoic acid.

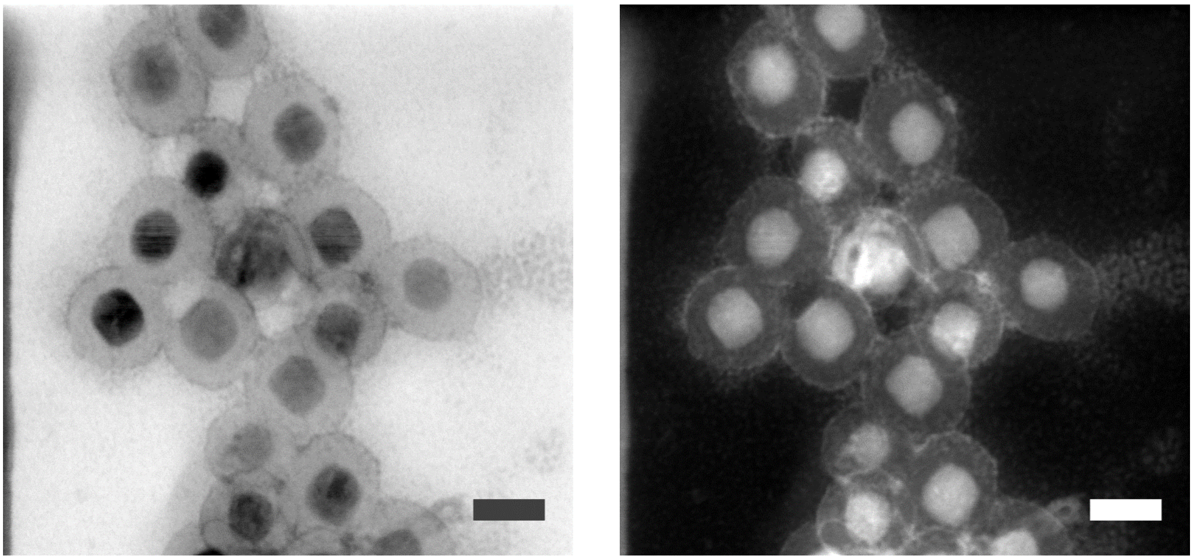

Comparison between bright-field and dark-field STEM images of the particles modifies using mercaptoundecanoic acid after coordination of Zn^{2+}, scalebar 20 nm.

Figure S9. Reference experiment: Treatment of unmodified Fe_3O_4/SiO_2/AlkySil with Zn^{2+}.

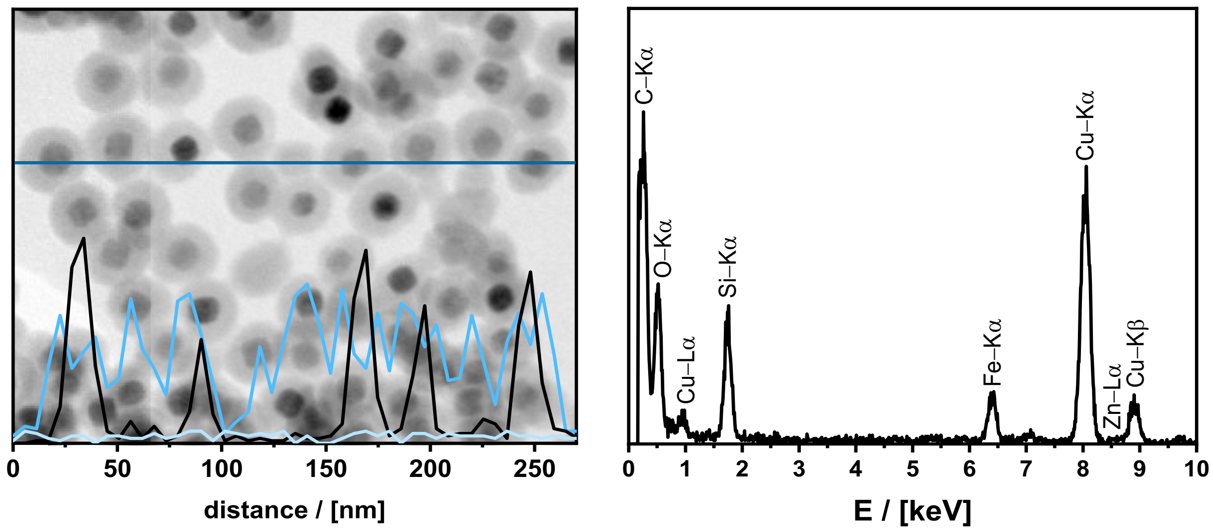

Left: STEM-micrograph, EDX line scan (black = Fe; blue = Si; light blue = Zn; dark blue: position of linescan), right: EDX spectrum. Reference experiment shows no coordinated Zn ions.

Figure S10. Monolayer of Fe_3O_4/SiO_2/AlkySil on a silicon substrate.

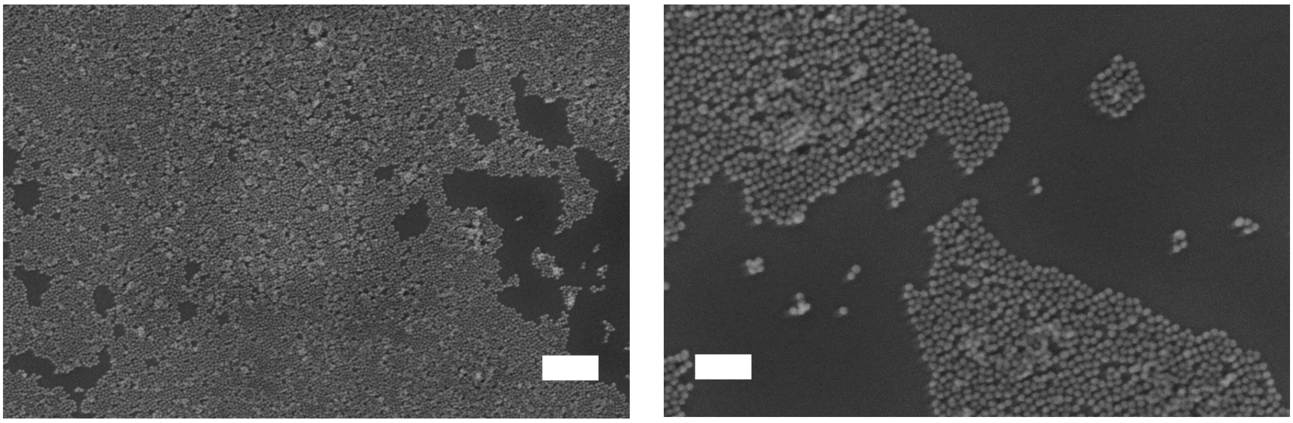

SEM micrographs, left: scalebar 500 nm, right: scalebar 200 nm.

Figure S11. Comparison of EDX spectra of Zn-modified particles.

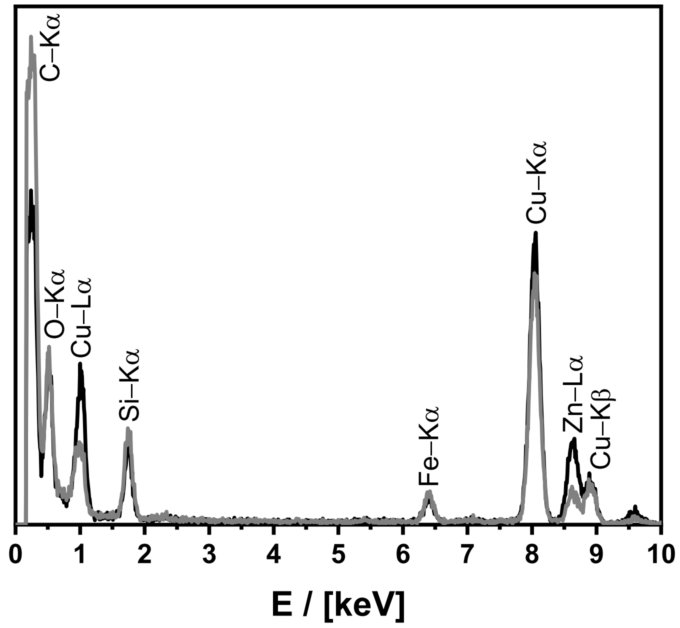

	Content of Si:Fe:Zn		
	**Si**	**Fe**	**Zn**
Fe_3O_4/SiO_2/AlkySil	2.9	1	-
Isotropic: Fe_3O_4/SiO_2/AlkySil/Zn	3.1	1	0.3
Anisotropic: Fe_3O_4/SiO_2/AlkySil/a-Zn	2.8	1	0.1

EDX spectra of isotropic-clicked Fe_3O_4/SiO_2/AlkySil/Zn (black) and anisotropic-clicked Fe_3O_4/SiO_2/AlkySil/a-Zn particles (grey). Referenced to Fe-signal; spectrum of isotropic-clicked particles have a higher content of Zn than the anisotropic-clicked particles. The ratio of Si:Fe:Zn are calculated from the measured at.-% given by the EDX spectra.

Figure S12. Analytical data for the click modification of the Fe_3O_4/SiO_2/AlkySil particles by the photochemical Thiol-Yne reaction: pentafluorothiophenol.

(a)

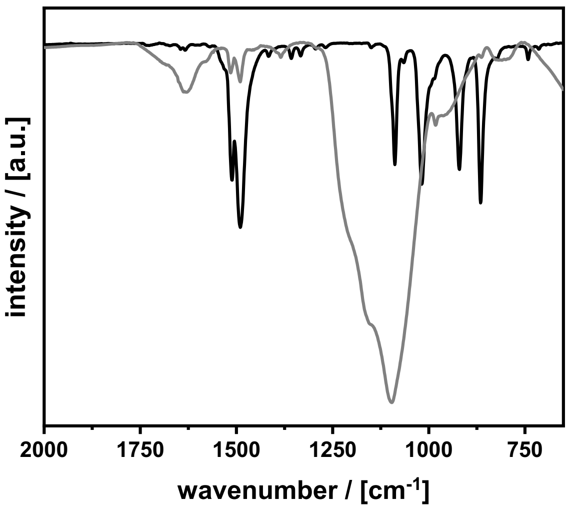

(a) IR spectra of Fe_3O_4/SiO_2/AlkySil/F-thiol (grey), pentafluorothiophenol (black). By comparing the IR spectra of pentafluorothiophenol (black) and the fluoro-modified core-shell-shell particles (grey) we see the characteristic C-F vibration band at 1488 cm^{-1}.

(b)

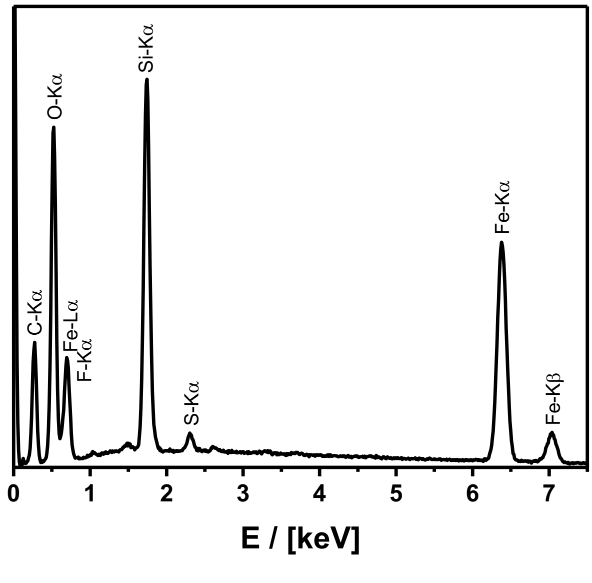

EDX spectrum of Fe_3O_4/SiO_2/AlkySil/F-thiol particles of the click reaction with pentafluorothiophenol.

Figure S13. Analytical data for the anisotropic click modification of the Fe_3O_4/SiO_2/AlkySil particles by the photochemical Thiol-Yne reaction with pentafluorothiophenol.

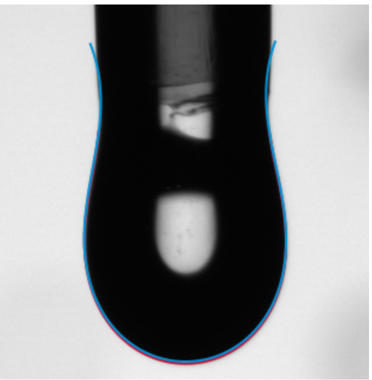

Pendant drop method for calculation of the surface energy of the the Fe_3O_4/SiO_2/AlkySil/a-F-thiol particle dispersion in ethanol, γ_{lv} = 20.4 mN/m.

Figure S14. Analytical data of contact angle measurements.

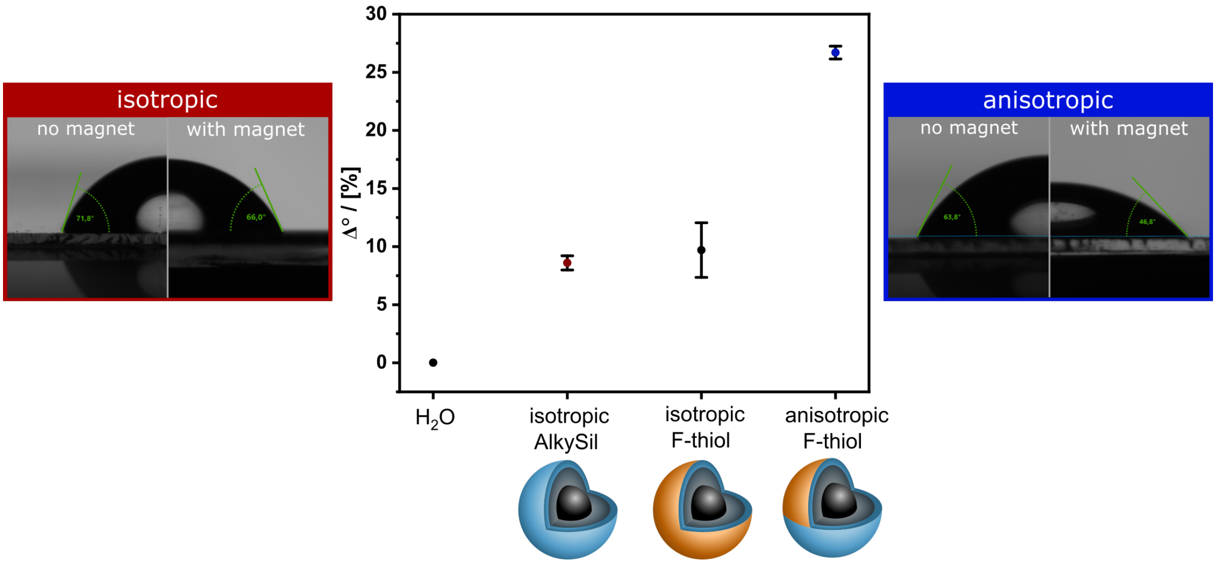

Control experiment of the change in contact angle measurements (Δ° in %) with water, Fe_3O_4/SiO_2/AlkySil (left images) and isotropic-clicked F-thiol particles in comparison with the F-thiol JPs (right images) without and with a magnet.

The contact angle of water stays constant with and without the magnet, whereas the measurements with the Fe_3O_4/SiO_2/AlkySil and isotropic-clicked F-thiol particles shows a change after applying a magnetic field. However, in both control experiments with isotropic particles the decreasing of the contact angle is obvious less than in the case of the JPs.
